# Squamous Cell Carcinoma Arising From a Mature Cystic Teratoma of the Ovary: A Case Report

**DOI:** 10.7759/cureus.12846

**Published:** 2021-01-21

**Authors:** Andres Rave Ramirez, Octavio Arencibia-Sánchez, Miguel Andújar Sánchez, Alicia Martin Martinez, Maria Laseca-Modrego

**Affiliations:** 1 Gynecologic Oncology, Complejo Hospitalario Universitario Insular-Materno Infantil, Las Palmas de Gran Canaria, ESP; 2 Pathology, Complejo Hospitalario Universitario Insular-Materno Infantil, Las Palmas de Gran Canaria, ESP

**Keywords:** squamous, carcinoma, mature, teratoma, ovary

## Abstract

Germ cell tumors represent 20-25% of ovarian tumors, and 95% of them are benign. The most frequent type is the mature benign teratoma (dermoid cysts). The proportion of cases in which malignancy occurs is 0.17-2%. Seventy-five percent to 90% of malignancies are squamous cell carcinomas (SCC).

We present a case of squamous cell carcinoma originating from a mature cystic teratoma that was diagnosed after intraoperative pathology study in a 64-year-old woman who consulted for an adnexal tumor causing abdominal pain. Laparoscopic surgery was scheduled, describing an enlarged right ovary (13 cm) which was included in the ipsilateral broad ligament and adhered to the posterior aspect of the uterus in its distal third as well as the rectum. It was converted to laparotomy and we performed a hysterectomy + double anexectomy + omentectomy + resection of sigma with end-to-end anastomosis after intraoperative pathological study reported for malignancy compatible with squamous cell carcinoma. It was labeled as FIGO III stage. Chemotherapy was decided as adjuvant therapy with carboplatin + paclitaxel (Carbo-Taxol) scheme.

We review the existing literature to provide evidence on a rare pathology with important repercussions for our patients.

## Introduction

Between germ cell tumors of the ovary, the most frequent tumor line is the mature benign teratoma, also known as dermoid cysts. Mature cystic teratomas (MCT) are very frequent in women of reproductive age and almost 10-17% of the time they present bilaterally. They can be formed by mature tissues from the three germ layers (ectoderm, mesoderm, and endoderm). Most patients with mature teratomas are asymptomatic but may develop pain and a feeling of abdominal occupation due to the mass effect [1].

Although mature teratomas are usually benign, malignancy can occur and is more common in older, postmenopausal patients. The reported incidence of mature teratomas is 1.2-14.2 cases per 100,000 per year, and the proportion of cases in which malignancy occurs is 0.17-2%. Between 75-90% of the malignancies are squamous cell carcinomas (SCC) [2]. Adenocarcinomas, carcinoid tumors, melanomas, sarcomas, and neuroepithelial tumors have also been reported.

Preoperative diagnosis of malignancy is very difficult because symptoms and signs are nonspecific, and neither tumor markers nor imaging techniques can predict the specific diagnosis [3]. Regarding clinical manifestations, tumors in early stages are usually detected accidentally during a physical examination or after postoperative pathological analysis, while a palpable mass, bloating, and abdominal pain usually occur in more advanced stages. It could also present as an acute abdomen due to torsion or rupture of the tumor [4].

Regarding treatment, it is also not clear which is the best approach. Some argue that since SCC originates from the epithelium, its treatment should follow the principles of epithelial ovarian cancer. Others believe that treatment should follow SCC guidelines from other origins, and finally there are those who suggest that since malignancy arises in the context of a mature teratoma, treatment should be according to protocols for ovarian germ cell tumors. The problem is that these three approaches are totally different [4,5].

The objective of this article is to present a case of squamous malignancy over a mature teratoma that was diagnosed after the intraoperative pathology study.

## Case presentation

Our patient is a 64-year-old woman, who presented with an adnexal mass with features suggestive of a malignant tumor. The patient presented with abdominal pain, which is why an abdominal ultrasound was performed. She had no constitutional syndrome (asthenia, anorexia, and/or involuntary weight loss).

She had no relevant pathological family history. She only suffered from hypertension controlled with medication and had undergone an appendectomy. Regarding gynecological history, menarche at 13 years, G2P2, menopause at 53 years. She did not refer previous gynecological controls. No toxic habits.

**Figure 1:**
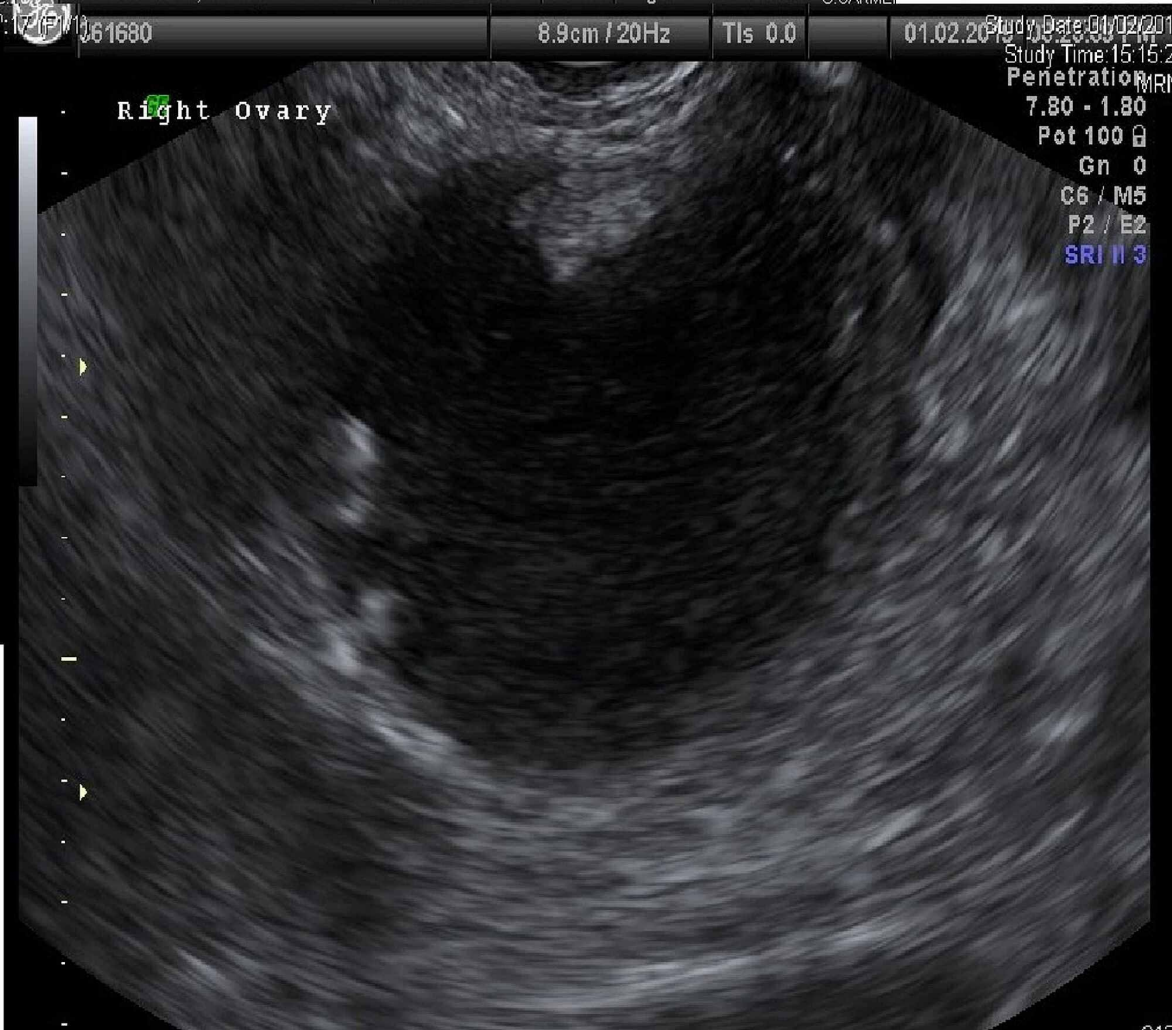
Transvaginal ultrasound: Adnexal cyst with papillary formation

**Figure 2:**
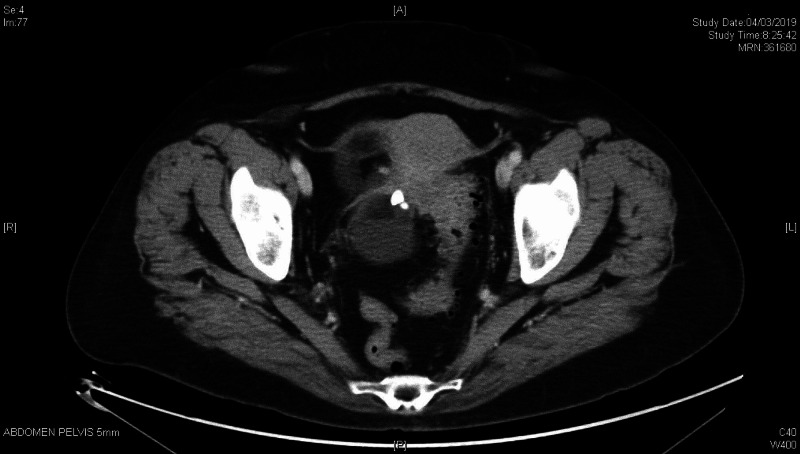
CT scan Right adnexal lesión; size 7.9x10.4x11.9 cm, presence of fat and calcium content inside, without solid pole images, suggestive of teratoma

In transvaginal ultrasound and computarized tomography (Figures 1, 2), a uterus in anteversion was observed, enlarged in relation to two myomatous nodules with hypoechogenic appearance, both intramural, one 26x17x29 mm in the fundus and posterior aspect and another 30x23x31 mm in the anterior aspect; 2 mm linear endometrium.

Right ovary, formation with the following characteristics: size 77x65x103 mm. Solid unilocular. Mixed content, reminiscent of the dermoid cyst (although the vascularized solid región impresses more solid than a typical dermoid hyperechogenic ball). Regular contour. Irregular wall at the expense of a solid 73x73 mm region, vascularized. Doppler color score type 3. Rear acoustic shadow is observed. No healthy ovarian tissue is observed. Lack of fluid in Douglas. Normal kidneys, absence of perihepatic or perisplenic fluid. The evaluation of lymph nodes is limited due to the adiposity presented by the patient. The only striking data is the presence of a 15 mm thickening of the greater omentum, vascularized. IOTA Adnex score (no Ca125): 36.5%, at the expense of stage I primary tumor. Diagnostic suspicion: right adnexal tumor, with echographic signs suggestive of malignancy. Negative tumor markers (carcinoembryonic antigen [CEA] 1.3; cancer antigen 125 [CA125] 6.1; CA19.9 0.8; alphafetoprotein 7.3; human epididymis protein 4 [HE4] 79.3)

**Figure 3:**
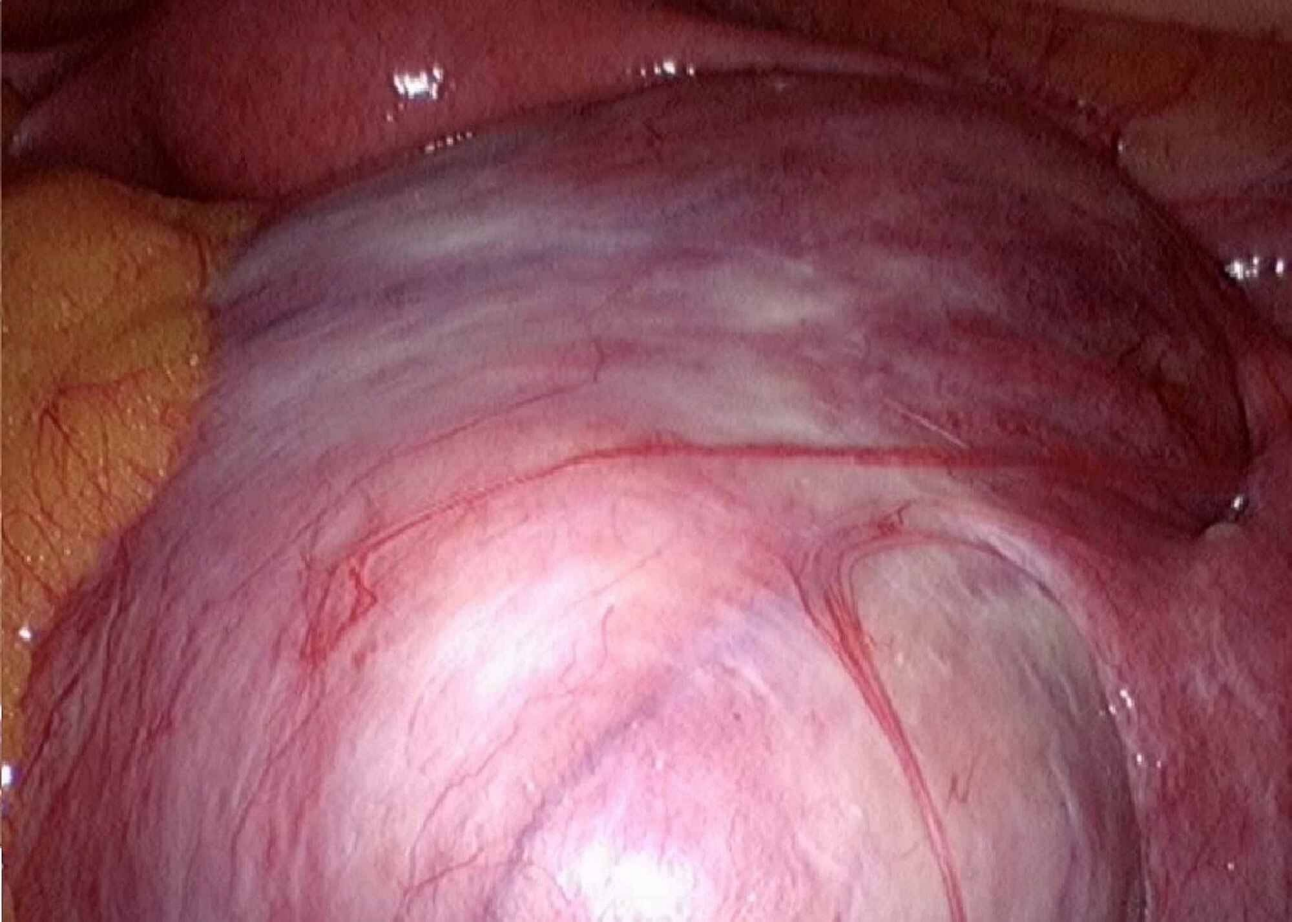
Laparoscopic view of a right ovary tumor

She was scheduled for surgery, which was started laparoscopically, describing a normal uterus, a right ovary enlarged at 13 cm, with a smooth pearly-looking capsule, which was included in the ipsilateral broad ligament and adhered to the posterior aspect of the uterus in its distal third as well as the rectum (Figure 3). Left ovary presented with normal appearance, with adhesions to epiploic appendages from the tube. Tumor is observed at the sigma level. Intestine, omentum, peritoneum, small intestine, diaphragmatic domes, liver, and stomach without implants.

**Figure 4:**
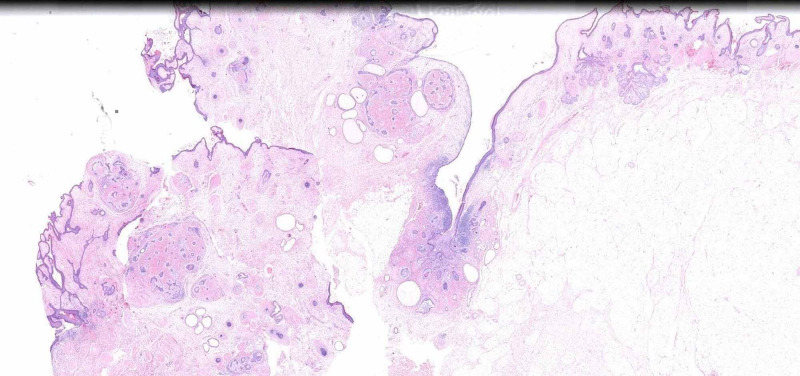
Panoramic view of mature teratoma with the presence of superficial squamous epitelial tissue, skin appendages and fat

**Figure 5:**
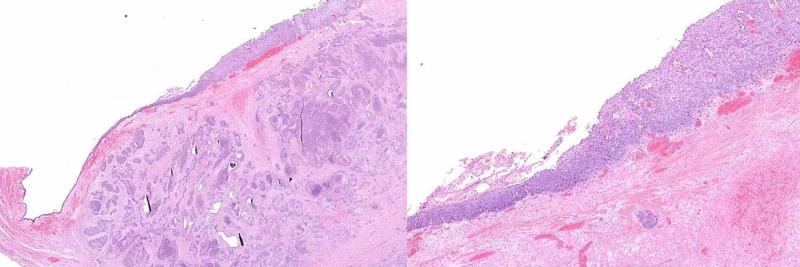
Panoramic histological preparation showing the squamous epithelium of the carcinoma “in situ” on the surface and next to it the infiltrating component formed by irregular nests

**Figure 6:**
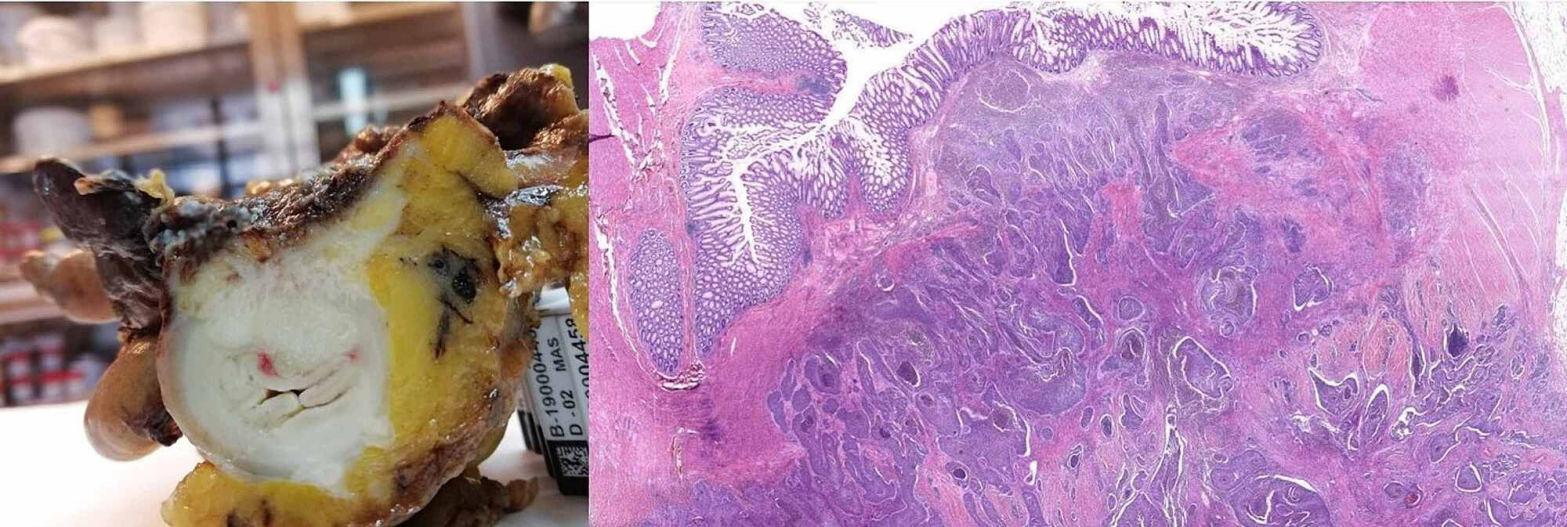
Image A (Right): cross section of the colon wall where the infiltration of the tumor in the upper and central part is observed. Image B (left): Histological panoramic view of the cross section of the colonic wall: the upper part shows the mucosa and the underlying infiltrating tumor composed of irregular solid nests.

It was converted to laparotomy and we performed a hysterectomy + double anexectomy + omentectomy + resection of sigma with end-to-end anastomosis after intraoperative pathological study reported positive for malignancy compatible with squamous cell carcinoma. Incidences in the surgery were not described. The definitive pathology report describes moderately differentiated infiltrating squamous cell carcinoma originating from mature ovarian cystic teratoma. Piece infiltrates intestinal wall respecting mucosa. Omentum was negative for malignancy (Figures 4, 5, 6). It was labeled as FIGO III stage.

Patient was presented in multidisciplinary committee, where chemotherapy was decided as adjuvant therapy. She received Carbo-Taxol scheme every 21 days x six cycles, with good tolerance to treatment, only presenting grade II alopecia, without significant neutropenia.

## Discussion

The most recent systematic review published in the literature (2018), carried out by Li et al., analyzed data from 63 cases of SCC on MCT published between 1977 and 2016, and obtained very important conclusions. In this study, the mean age at diagnosis was 53.5 years (19-87), with a clear predominance of patients in the group >45 years (27.9% vs. 72.1%), with a worse prognosis in this group. This result is statistically significant. The mean tumor size was 14.8 cm (3.5-40 cm). Mortality of 40.2% was estimated. There was no significant difference in survival between tumors <10 cm and >10 cm. Abdominal pain and palpable abdominal mass were the two most frequent clinical manifestations in SCC malignancy, occurring in 47.3% and 26% of all cases. Regarding the stage at diagnosis, stages I, II, III, IV presented in 50%, 18.8%, 26.8%, and 4.4% respectively, with stages II, III, and IV having a worse prognosis compared to stage I in a significant way. The overall survival at five years was 85.8%, 39.1%, 26.2%, and 0% for stages I, II, III, IV, respectively [4].

Regarding treatment, comprehensive staging surgery is the standard for ovarian cancer. It was found that in SCC on MCT, hysterectomy can reduce the risk of death in a statistically significant way, whereas lymphadenectomy does not improve survival (however, given the retrospective nature of this review, the authors recommend that it should be performed as part of a comprehensive staging surgery). Omentectomy showed an increase in survival when a FIGO stage adjusted analysis was performed. There were no differences in mortality in patients (24) in whom fertility-sparing surgery was performed versus radical surgery in stages IA-IC <45 years [4].

There is no consensus on which is the first line in adjuvant treatment in patients with SCC over MCT because of the rarity of this entity. Adjuvant chemotherapy may improve survival in patients with advanced disease, and platinum chemotherapy had better results than other compounds in this study [4]. Previously, Hackethal et al. in their 2008 systematic review suggested that alkylating agents had better results. Overall survival of patients had improved to 57.1 months when alkylating agents were administered compared to 25.2 months for those who received non‐alkylating regimens [1]. A higher survival was associated with complete resection followed by adjuvant chemotherapy in advanced disease but adjuvant radiotherapy did not improve survival of the patients in the same study. Cisplatin-ifosfamide-paclitaxel (TIP), a highly active chemotherapy regimen, has proven to be efficient in SCC arising in MCT. The combination of carboplatin-paclitaxel regimen has also been reported. Radiotherapy might actually lead to greater morbidity and adverse survival [1].

Li et al. could not analyze the data prior to diagnosis due to the retrospective nature and the lack of data exposed in the different publications on which it is based [4]. However, it is important to identify the presence of malignancy associated with mature teratoma to determine the surgical approach. Radiological findings such as the presence of a solid component, known as the Rokitansky bulge, transmural extension or invasion of adjacent tissues, and biochemical findings such as elevation of SCC, CA125, CA19.9, and CEA antigen may help in predicting malignancy. Likewise, a Chinese study found human papillomavirus (HPV) infection in a series of four cases of SCC on MCT, opening the hypothesis that HPV infection may be a risk factor to take into account in this type of patient [6]. Although the intraoperative pathology study is not the gold standard for patients with this type of pathology who undergo routine surgery, it should be considered in certain patients, such as postmenopausal patients [7,8]. The predictive value of the intraoperative study for the detection of malignancy in MCT is 100%, with a sensitivity of between 60-100% [2]. Other studies tried to identify the genomic abnormalities in this type of tumor and evidenced that it has a high mutational burden, with TP53 mutation the most common abnormality. They found that the presence of TP53 mutation was a good prognostic factor [9].

## Conclusions

Malignancy in mature cystic teratomas is a rare entity. However, clinicians should suspect malignancy in mature cystic teratoma when the patient is postmenopausal, the tumor is giant and has large solid foci. The intraoperative pathological report is a valuable tool for detecting malignancy in these patients and should be used to assess therapeutic attitude. Diagnosis in early stages improves survival. Fertility-sparing treatment in patients with an unfulfilled desire for children does not worsen survival. There is no consensus regarding first-line drugs in adjuvant chemotherapy, but this treatment modality may improve survival on advanced stages when using alkylating agents. Adjuvant radiation therapy does not appear to affect survival.
